# Prognostic Value of FasL, BDNF, and IL-1β as Predictors of Therapeutic Response in Schizophrenia

**DOI:** 10.3390/jcm14186417

**Published:** 2025-09-11

**Authors:** Zofia Szymona-Kuciewicz, Maja Owe-Larsson, Marta Flis, Hanna Karakula-Juchnowicz, Barbara Zdzisinska, Ewa Dudzinska, Ewa M. Urbanska, Kinga Szymona

**Affiliations:** 1 Collegium Medicum, Cardinal Stefan Wyszynski University in Warsaw, Woycickiego 1/3, 01-938 Warsaw, Poland; zofia.szymona@gmail.com; 2 Department of Histology and Embryology, Center of Biostructure Research, Medical University of Warsaw, Chalubinskiego 5, 02-004 Warsaw, Poland; maja.owe-larsson@wum.edu.pl; 3 Center for Psychotherapy and Psychodynamic Psychiatry, Bernardynska 12/11, 20-109 Lublin, Poland; martaflis7@gmail.com; 4 Department of Psychiatry, Psychotherapy and Early Intervention, Medical University of Lublin, Gluska 1, 20-439 Lublin, Poland; hanna.karakula-juchnowicz@umlub.edu.pl; 5 Department of Virology and Immunology, Institute of Biological Sciences, Faculty of Biology and Biotechnology, Maria Curie-Sklodowska University, Akademicka 19, 20-033 Lublin, Poland; barbara.zdzisinska@mail.umcs.pl; 6 Department of Dietetics and Nutrition Education, Medical University of Lublin, Jaczewskiego 8b, 20-059 Lublin, Poland; ewa.dudzinska@umlub.edu.pl; 7 Department of Experimental and Clinical Pharmacology, Medical University of Lublin, Jaczewskiego 8b, 20-090 Lublin, Poland; 8 Institute of Medical Sciences, The John Paul II Catholic University of Lublin, Konstantynow 1 H, 20-708 Lublin, Poland; kinga.szymona@kul.pl

**Keywords:** schizophrenia, treatment-resistant, biomarker, inflammation, apoptosis, interleukin 1β, brain-derived neurotrophic factor, Fas ligand, clozapine

## Abstract

**Background/Objectives**: Pro-inflammatory, neurotrophic, and proapoptotic factors affect the course of schizophrenia; however, their impact on the clinical response during relapse is not well recognized. A member of TNF family, Fas ligand (FasL), participates in apoptosis, but its connection with treatment-resistant schizophrenia is unknown. **Methods**: For this preliminary exploratory study, 53 patients with schizophrenia relapse and 45 healthy subjects were enrolled. Pro-inflammatory interleukin IL-1β, brain-derived neurotrophic factor (BDNF), FasL levels, and clinical evaluations (PANSS, SANS, SAPS) were studied at admission, after a 4-week therapy, and at remission. **Results**: In the clozapine-treated therapy-resistant group, IL-1β correlated negatively with clinical improvement (admission, 4-week treatment). In patients not treated with clozapine, IL-1β correlated negatively with disease duration (admission). A negative correlation occurred between FasL and clinical improvement in general symptoms (admission, 4-week treatment), FasL and leukocyte count (admission), and IL-1β and BDNF levels (4-week treatment). In the clozapine-treated group, the negative correlation between FasL levels and the leukocyte count was absent. **Conclusions**: The severity of psychopathology in patients with schizophrenia seems to correlate with higher IL-1β and lower BDNF. The novelty of our findings is the observation that higher FasL is negatively associated with the degree of clinical improvement. Thus, a decline of FasL during treatment may be proposed as a predictor of clinical recovery. With caution, we suggest that clozapine use may be linked to a protective effect against FasL signaling and the alleviation of apoptotic processes.

## 1. Introduction

Schizophrenia spectrum disorders are complex psychiatric illnesses with a lifetime prevalence estimated at 0.5–0.7% [1]. Symptoms of schizophrenia, classically divided into positive, negative, and cognitive, comprise disturbed thought, self-experience, cognition, volition, affect, and behavior. Persistent delusions and hallucinations with disrupted thinking are principal manifestations of the disease [2].

With the progress of research, theories underlying the development of schizophrenia moved forward from a hypothesis of deranged neurotransmission, mostly dopaminergic and glutamatergic [3,4]. The pathogenesis of schizophrenia spectrum disorders involves a network of genetic predisposition, infectious factors, and stress-related changes. These, in turn, lead to aberrant brain development, dysfunctional synaptic activity, impaired neurogenesis, and distorted behavior and cognition [5,6].

Neuro-immune dysfunction is generally accepted as one of the major etiological factors in schizophrenia development. A disturbed immune response, at any ontogenetic stage, from the prenatal period until maturity, may increase the risk of disease. Furthermore, the balance between pro-inflammatory and anti-inflammatory responses is disrupted in schizophrenia, as evidenced by studies on peripheral blood and cerebrospinal fluid (CSF) [7,8,9]. The excessive activation of macrophages and T helper cells, with an excessive release of cytotoxic immune mediators, is often paralleled by a compensatory downregulation of immune response [10,11]. Altered cytokines can also be normalized in the course of treatment. High serum levels of interleukin 1β (IL-1β), IL-6, or tumor necrosis factor-α (TNF-α), often manifested during acute relapse of schizophrenia, are significantly reduced by antipsychotic therapy [9]. Some cytokines are increased only at the late stage of disease, but not in first-episode patients [12]. Conversely, other immunoactive molecules, such as α1-antitrypsin, B-lymphocyte chemoattractant, and IL-15, are negatively correlated with schizophrenia symptoms, both positive and negative, as measured by the Positive and Negative Symptom Scale for Schizophrenia (PANSS) scale [13]. Our previous studies have shown a negative association of serum interferon α (IFNα) and response to antipsychotic treatment in patients diagnosed with schizophrenia [14].

It is well established that a disturbed immune response may impair hippocampal neurogenesis. Altered neuronal development, changes in neurotrophic factor expression, and subsequent activation of apoptosis have been demonstrated in schizophrenia [15]. Among neurotrophins, brain-derived neurotrophic factor (BDNF) has been implicated in the pathogenesis of psychiatric illnesses [16,17]. There are three isoforms of BDNF expressed by neurons: the precursor form (proBDNF), the form converted by metalloproteinase-9 to the mature form (mBDNF), and the truncated form (truncated-BDNF). BDNF acts via the p75 neurotrophin receptor (p75NTR), and, in part, through the tropomyosin receptor kinase (Trk) receptor family. BDNF, along with neurotrophin-4 (NT-4), is a ligand for the tropomyosin receptor kinase B (TrkB) receptor [15,18,19]. TrkB receptor activation leads to Ras-mediated signal transduction, which is crucial for nerve cell maturation and neuronal plasticity [20]. The outcome of BDNF signaling varies depending on its isoform. mBDNF induces TrkB phosphorylation, whereas proBDNF interacts with p75NTR and sortilin and mediates cell death when those receptors are co-expressed [21,22]. Disturbed BDNF signaling is implicated in schizophrenia pathogenesis; however, the available data are not fully coherent [15,23]. Both decreased and unaltered BDNF levels were reported in schizophrenia [17].

In line with the neurodevelopmental theory of schizophrenia, emerging data implicated disturbed apoptosis in its pathogenesis. Changed apoptotic regulatory proteins and altered DNA fragmentation were reported in various cortical regions of patients known to have schizophrenia [24]. A member of the tumor necrosis factor (TNF) family, Fas ligand (FasL, CD95L) is involved in apoptosis of lymphocytes. The extrinsic apoptotic pathway starts with the binding of TNFα and FasL with their respective membrane receptors, TNFR1, TNFR2, and Fas (CD95) [25]. The Fas receptor consists of a transmembrane fragment and a cytoplasmic domain. The latter is referred to as the Fas-associated death domain (FADD). FADD binds the initiator caspase-8, thereby forming a death-inducing signal complex, whose activation leads to caspase-mediated apoptosis [26]. The roles of the FasL and Fas receptors in schizophrenia were only the subject of single reports, and the data are ambiguous [27,28].

Despite the quick development of novel antipsychotic agents, partial response or a lack of response to treatment are still high in schizophrenia. Moreover, resistance to therapy is associated with longer hospitalization and worse prognosis, negatively affects the functioning of the patient and their family, and brings significant social costs [29]. In treatment-resistant patients, clozapine remains the gold standard in therapy; however, the mechanisms underlying its efficacy are still not fully understood [30]. Reliable, easily measurable biomarkers may significantly improve the prediction of the response to therapy, and are especially sought for treatment-resistant patients. Moreover, the appropriate choice of biomarkers may facilitate personalized treatment tailored to a given patient, especially when combined with clinical tools [31,32].

There is a lack of clearly indicated correlations between the baseline status of pro-inflammatory, neurotrophic, and proapoptotic factors and clinical response during relapse of chronic schizophrenia. Furthermore, to our knowledge, there is no information about FasL in patients with treatment-resistant schizophrenia. Thus, we aimed to explore the baseline levels of IL-1β, BDNF, and FasL, and to assess their correlations with clinical status at admission, after a 4-week therapy, and at remission. Potential relationships between the studied parameters were explored, taking into consideration the disease duration and resistance to treatment.

Part of these data are presented as an abstract [Soluble Fas ligand (sFasL) as a predictor of the reduction in general psychopathology in schizophrenia after antipsychotic treatment] at the 24th European Congress of Psychiatry, Madrid, Spain, 2016 [33].

## 2. Materials and Methods

### 2.1. Study Group

The study group consisted of 98 individuals and included 53 patients aged 16–54 years diagnosed with schizophrenia according to the ICD-10 classification (1997; used at that period to classify patients), who were hospitalized in the Department of Psychiatry, Medical University of Lublin, in the years 2012–2014, and 45 healthy subjects, matched for age and education. The study protocol was approved by the Bioethics Committee at the Medical University of Lublin (approval no. KE-0254/77/2012) and was in agreement with the Helsinki Declaration of 1975.

Patients were recruited consecutively from admissions to the Department of Psychiatry, Medical University of Lublin, between 2012 and 2014, according to predefined inclusion and exclusion criteria. All eligible individuals who met the diagnostic requirements and provided written informed consent were included in the study.

In total, 72 patients diagnosed with schizophrenia were initially enrolled. The inclusion criteria were as follows: clinical diagnosis of schizophrenia according to ICD-10 (1997), informed consent to participate in the study, and age between 16 and 54 years. The exclusion criteria were as follows: active or past neurological diseases, infectious diseases before admission (1 month) or during hospitalization, autoimmune diseases, severe chronic diseases such as diabetes or cardiovascular diseases, anti-inflammatory drug intake in the 2 weeks preceding/during hospitalization, alcohol abuse, smoking more than 10 cigarettes a day in the last 3 months), intellectual disability or dementia. In total, 53 people diagnosed with schizophrenia were selected for further studies and divided into the following groups: (A) According to the duration of the disease: (1) up to 12 months: 1st stage, early-phase schizophrenia (*n* = 16); (2) between 13 and 60 months: 2nd stage (*n* = 19); (3) over 60 months: 3rd stage, chronic schizophrenia (*n* = 18). (B) According to the treatment: (1) clozapine-treated (*n* = 11), (2) non-clozapine treated, receiving other antipsychotics (*n* = 42), including olanzapine, aripiprazole, fluphenazine and risperidone.

The control group included 45 individuals, unrelated to each other or to the patients from the study group, who met the following conditions: age between 16 and 54 years, and informed consent to participate in the study. Exclusion criteria were the same as for the study group.

Patients were evaluated at admission, after 4 weeks of hospitalization, and at remission, 1 week before discharge from the hospital. At admission, a psychiatric interview and a physical examination were performed, and written consent to participate in the study, including its scientific use and publication in anonymized form, was obtained.

### 2.2. Plasma IL-1β, FasL, and BDNF Concentration Measurements

Blood sampling for plasma measurement of BDNF, FasL, and IL-1β were performed at admission, after 4 weeks of hospitalization, and at remission.

The collected blood samples were centrifuged for 10 min at 2000 rpm and stored at −72 °C. IL-1β, FasL, and BDNF concentrations were determined in the plasma using the enzyme-linked immunosorbent assay (ELISA), using kits from R&D Systems (Minneapolis, MN, USA), at the Department of Immunology, Maria Curie-Skłodowska University in Lublin. The detection level of the determined substances was as follows: 1.20 pg/mL for IL-1β, 2.66 pg/mL for FasL, and 20.00 pg/mL for BDNF.

### 2.3. Mental Status Assessment

The evaluation of mental status was performed using the following scales: Positive and Negative Syndrome Scale (PANSS), Scale for Assessment of Positive Symptoms (SAPS), and Scale for Assessment of Negative Symptoms (SANS). The PANSS is a standardized, clinician-rated instrument comprising 30 items, grouped into three subscales: positive (7 items), negative (7 items), and general psychopathology (16 items). Items are rated from 1 (absent) to 7 (extreme). PANSS demonstrates high internal consistency (Cronbach’s α ranging from 0.73 to 0.83 for the three subscales) and good inter-rater reliability (intraclass correlation coefficients from 0.83 to 0.87). It also shows good construct and criterion validity in patients diagnosed with schizophrenia [34,35,36]. The total score is calculated by summing the scores on the three subscales. It is reported separately for negative symptoms (PANSS N), positive symptoms (PANSS P), global symptoms (PANSS G), and the total score (PANSS T).

The SAPS and SANS scales were constructed by Nancy Andreasen [37]. SAPS evaluates 35 positive symptoms, divided into 4 groups: delusions, hallucinations, bizarre behavior, and formal thought disorders. SANS includes 24 negative symptoms, divided into 5 groups: affective blunting, alogia, avolition and apathy, anhedonia and asociality, and attention disorders. Positive and negative symptoms can be diagnosed when a symptom occurs in all domains. The severity of symptoms is determined in a score from 0 to 5: 0—no symptom, 1—plus–minus, 2—minimal, 3—mild, 4—severe, 5—significantly severe.

The effectiveness of antipsychotic drug therapy was assessed by calculating the percentage reduction in symptom severity on the PANSS, SAPS, and SANS scales. The percentage reduction in symptom severity ∆ (improvement in clinical symptoms) was calculated according to Leucht [38]. In clinical scales, treatment response is defined as a reduction in total scores of at least 25%. PANSS percentage change (∆PANSS) measures the percentage of patients with a good treatment response who have a percentage reduction in PANSS clinical scale scores of >25%. The associations between the degree of clinical improvement (PANSS percentage change), defined in the paper as ∆PANSS, ∆SAPS, and ∆SANS, and the studied markers were determined to find predictors of clinical improvement in terms of positive, negative, and general symptoms, as well as in groups divided according to the type of antipsychotic treatment, namely the group of patients treated with clozapine and the group of patients treated with other antipsychotic drugs.

### 2.4. Statistical Analysis

Statistical analyses were performed using Statistica 10 for Windows. The mean and standard deviation SD were calculated for all parameters. The normality of distribution was checked using the Shapiro–Wilk test for all variables. The Mann–Whitney U test was used to compare two independent samples, and the Wilcoxon signed-rank test was used to compare the differences between three measurements at admission, during treatment, and in remission. The Spearman rank correlation coefficient was used to assess the strength of correlations between subsequent parameters. Correlations between the tested substances and the intensity of psychopathology and the degree of clinical improvement in the PANSS, SAPS, and SANS scales were examined. Values with the coefficient equal to * *p* < 0.05 were considered statistically significant.

Post hoc statistical power analysis was performed for the primary correlation analyses. In the whole patient group (*n* = 53), this study had >80% power to detect Spearman correlation coefficients with |r| ≥ 0.37 at α = 0.05. In the clozapine-treated subgroup (*n* = 11), the power exceeded 80% for |r| ≥ 0.70, but was lower for weaker associations. Given these constraints, the results from small subgroups are considered exploratory, and are interpreted with caution.

To control for multiple comparisons in correlation analyses, the Benjamini–Hochberg false discovery rate (FDR) procedure was applied with a significance threshold of q = 0.05. FDR correction was performed separately for each correlation table (Tables S4–S9) using exact *p*-values calculated from Spearman’s *r* and group sample sizes.

## 3. Results

### 3.1. Group Characteristics

The patients participating in this study were aged 16–54 years, while the age of the healthy controls ranged between 17 and 49 years. The patient group was dominated by men (64.15%). Most of the patients known for schizophrenia had secondary education (41.51%), and a fairly large number of them had higher education (32.08%). There were no statistically significant differences in the leukocyte count and body mass index (BMI) between the patients and controls. Detailed data on group characteristics are presented in Table 1. In the patient group, the average duration of psychosis was 70.00 months (5.8 years) ± SD 73.0. The disease variables are demonstrated in Table 2.

### 3.2. Clinical Schizophrenia Scale Scores and IL-1β, BDNF, and FasL Levels

The patients’ mean total scores in the PANSS scale (PANSS T) were 92.0 ± 21.7, the mean positive symptom score (PANSS P) was 21.9 ± 6.6, the mean negative symptom score (PANSS N) was 25.0 ± 7.3, and the general symptom score (PANSS G) was 45.0 ± 12.43. The severity of symptoms measured using the clinical scales PANSS, SANS, and SAPS decreased significantly under the influence of antipsychotic treatment during hospitalization. There were no significant differences between the levels of IL-1β, FasL, or BDNF in the plasma of patients diagnosed with schizophrenia and the healthy control group (Table 3).

### 3.3. Correlations Between IL-1β Levels and PANSS, SAPS, and SANS Scores

In the group of all studied patients, IL-1β level correlated with the severity of symptoms, as shown by the PANSS N (r = 0.30) and SANS (r = 0.44) scales. A negative correlation (r = −0.40) was demonstrated between ∆PANSS P and IL-1β after 4 weeks of treatment. In the clozapine-treated group, a strong negative correlation (r = −0.86) was demonstrated between ∆PANSS P and IL-1β at admission and after 4-week treatment. In the same group, a negative correlation was demonstrated between ∆PANSS T and IL-1β after 4-week treatment. No significant correlations were found between IL-1β and the number of relapses, leukocyte count, and disease duration in the clozapine-treated group. In the group without clozapine treatment, IL-1β levels correlated negatively with disease duration at admission (r = −0.41) (Supplementary Materials, Tables S1 and S2).

### 3.4. Correlations Between FasL Levels and PANSS, SAPS, and SANS Scores

In the entire group of patients, FasL level correlated negatively with the degree of clinical improvement in general symptoms (admission and 4-week hospitalization) (r = −0.39 and −0.43, respectively), and with leukocyte count at admission (r = −0.33). In the clozapine-treated group, FasL level correlated negatively with the degree of clinical improvement (ΔPANSS T) after 4-week therapy (r = −0.68) and with ΔPANSS G at admission and after 4-week therapy (r = −0.77 and −0.70, respectively). In the group not treated with clozapine, there was a negative correlation between FasL and leukocyte count at admission (r = −0.43) (Supplementary Materials, Tables S3 and S4).

### 3.5. Correlations Between BDNF Levels and PANSS, SAPS, and SANS Scores

There were no significant correlations between BDNF and clinical scales, degree of clinical improvement, and disease variables in all studied groups (Supplementary Materials, Tables S5 and S6).

### 3.6. Correlations Between IL-1β and FasL/BDNF

In the group of all patients, a negative correlation was observed between IL-1β and BDNF after 4-week therapy (r = −0.48), and a positive correlation was found between the level of FasL and BDNF at admission (r = 0.34). In patients not-treated with clozapine, IL-1β correlated negatively with BDNF levels (r = −0.57) (Supplementary Materials, Table S7).

### 3.7. Correlations Between IL-1β, FasL, and BDNF Levels and Clinical Schizophrenia Scores in Patients with Early-Phase Schizophrenia

In the group of patients with early-phase schizophrenia (up to 1 year), positive correlations were found between IL-1β and the intensity of negative symptoms (measured using the PANSS N and SANS scales) after 4-week treatment (r = 0.83 and 0.73, respectively). A negative correlation (r = −0.72) was demonstrated between ∆ PANSS G and IL-1β after 4-week therapy. After 4-week treatment, BDNF correlated negatively with the intensity of negative symptoms (SANS) (r = −0.79), and positively with the degree of clinical improvement in general PANSS G symptoms (r = 0.78) (Supplementary Materials, Table S8).

### 3.8. Correlations Between IL-1β, FasL, and BDNF Levels and Clinical Schizophrenia Scores in Patients with Chronic Schizophrenia

In patients with chronic schizophrenia (over 5 years), IL-1β correlated negatively with ∆PANSS G and ∆PANSS T at remission (r = −0.82 and −0.9, respectively). Moreover, the following negative correlations were demonstrated: FasL with ∆PANSS T (r = −0.75 at admission and r = −0.72 after 4-week treatment), FasL with ∆PANSS G (r = −0.78 at admission and r = −0.91 after 4-week treatment), and FasL with ∆SANS (r = −0.62 at admission). BDNF concentrations correlated negatively with PANSS T and PANSS G scores after 4-week treatment (r = −0.75 and −0.69, respectively). BDNF levels were correlated positively with disease duration after 4-week therapy and at remission (r = 0.74 and 0.70, respectively) (Supplementary Materials, Table S9).

## 4. Discussion

In our study, the levels of IL-1β, Fas, or BDNF did not differ between patients suffering from schizophrenia and healthy controls; however, significant correlations were found between the studied markers and clinical symptom severity, disease duration, the type of treatment. Due to the relatively small sample sizes in some subgroups, the results concerning patients with treatment-resistant schizophrenia are preliminary.

The data on IL-1β are consistent with a meta-analysis from 2023 (patients diagnosed with schizophrenia *n* = 430, and healthy controls *n* = 346) [39] and with later studies [40,41,42]. No significant differences in serum IL-1β levels were found in patients with first-episode schizophrenia (*n* = 93) compared to healthy controls (*n* = 60) [40]. Similarly, others observed unaltered IL-1β in patients known for schizophrenia [41,42]. On the other hand, the expression of IL1-β was increased in human pluripotent cells derived from schizophrenic patients and later differentiated into neurons [43], as well as in the peripheral blood of patients diagnosed with schizophrenia [44].

The negative correlation we have demonstrated here between pro-inflammatory IL-1β levels and illness duration may imply that the subtle inflammation undergoes a gradual decline in the course of disease, possibly as a result of antipsychotic treatment. Other studies, however, found a significant positive correlation between IL-1β and total disease duration (r  =  0.638, *p*  =  0.006) [44]. The differences between studies may result from the heterogeneity of schizophrenia spectrum disorders, various pharmacological interventions, and different sizes of studied groups. In our study, no significant changes in IL-1β concentration after 4-week hospitalization and during remission were revealed in the group of all patients. However, some reports, including a meta-analysis, demonstrated an increased IL-1β concentrations in patients suffering from schizophrenia, which was normalized under the influence of antipsychotic treatment [9,45,46]. In contrast, others have shown that IL-1β levels rise in clozapine-treated women [47].

The positive correlation between plasma IL-1β levels and the severity of psychopathology in the PANSS N scale (during relapse) and SANS scale (after 4 weeks of therapy), observed here, is consistent with other reports. The degree of psychopathological changes in patients with schizophrenia or depression was linked with the levels of the following pro-inflammatory cytokines: IFN-α, IFN-γ, IL-1β, IL-6, and TNF-α [46,48]. A positive correlation between PANSS scores and serum IL-1β levels has also been reported [49]. Moreover, miRNAs (related to PANSS P, PANSS N, and PANSS T) were associated with inflammation and IL-1β [50]. High levels of IL-1β correlated with the severity of the depressive and negative symptom dimensions at first-episode psychosis [51]. IL-1β mRNA positively correlated with PANSS T scores and the PANSS G sub-scores [44].

In the group of patients with short-term illness, positive correlations were found between IL-1β and the severity of negative symptoms (measured with the PANSS N and SANS scales) after a 4-week therapy. Based on these results, it can be suggested that low IL-1β levels may be associated with greater treatment efficacy, particularly concerning negative symptoms. Similar correlations occurred in the group of chronically ill patients. Thus, a decrease in IL-1β during treatment may be a marker of clinical improvement in schizophrenia. Indeed, genetic studies have demonstrated changes in the *IL1B* gene in schizophrenia and a significant association of the *IL1B* gene polymorphisms with the risk of schizophrenia and age of onset [52].

There were no significant differences in the level of plasma FasL in the entire studied group compared to the control. The literature data on FasL in schizophrenia are very limited and equivocal. In clinical studies, blood FasL levels was either unaltered or higher [27,28], whereas *FAS* gene polymorphisms was not changed [53]. In an animal model of schizophrenia, there was no change in levels of Fas receptor and the adaptor protein FADD in cortical area [54]. The levels of the adaptor protein FADD and FADD mRNA were not altered in the brains of patients with schizophrenia [55]. In contrast, mRNA expression of the Fas ligand gene (*FASLG*) was decreased in patients with acute schizophrenia (*n* = 32) compared to healthy controls (*n* = 32) [56].

A novel finding of our study is a negative correlation between the level of FasL and the degree of clinical improvement in general symptoms (ΔPANSS G) at admission and after a 4-week treatment in the whole study group, as well as in the clozapine-treated group. Others reported that the mRNA expression of the *FASLG* was decreased after 4-week olanzapine treatment [56], and the level of FasL decreased after 6-week therapy with olanzapine treatment [28]. Thus, we carefully propose that a decrease in plasma FasL levels during treatment may be a predictor of clinical improvement in schizophrenia.

In studied here entire cohort of patients, as well as in the clozapine-free subgroup, a negative correlation between the FasL level and the leukocyte count was found. This observation suggests the potential role of FasL in the apoptotic processes among patients with schizophrenia. Considering that such correlation was not observed in the group of patients with treatment-resistant disease, clozapine seems to be associated with the reduction in leukocyte apoptosis. Further studies assessing a larger number of apoptotic factors involved in this process and examining the relationships between apoptotic factors in the blood and morphological changes in the brain are needed.

In the group of patients with chronic schizophrenia, FasL correlated negatively with the clinical improvement and with the degree of reduction in general symptoms, at admission and after 4-week therapy. Interestingly, none of these correlations occurred in the early-phase group, suggesting that development of schizophrenia may be linked, at least in part, with stimulation of apoptotic processes. It is consistent with other data showing that clozapine and olanzapine enhanced the expression of the antiapoptotic protein Bcl-2 in an animal model of schizophrenia [57]. Moreover, antipsychotic treatment increased the cortical expression of antiapoptotic factor, Bcl-2, in patients suffering from schizophrenia [58].

The lower the plasma level of the apoptosis factor FasL in patients suffering from schizophrenia for >5 years, the greater the degree of clinical improvement, particularly concerning general and negative symptoms. The potential value of FasL as a predictor of clinical improvement in schizophrenia requires confirmation in further studies.

The results obtained in this study showed no significant differences in plasma BDNF levels compared to the healthy control group. The available data on BDNF levels in schizophrenia are not consistent. So far, reductions, increases, and the lack of changes in BDNF levels have been reported, and there is no consensus on the usefulness of BDNF as a schizophrenia biomarker or a predictor of response to therapy [17,59].

In the studied cohort, a negative correlation between IL-1β and BDNF was observed after 4 weeks of hospitalization in the group of all patients and among patients not treated with clozapine. This suggests that higher levels of pro-inflammatory IL-1β may negatively impact neurotrophic signaling. Indeed, neuroinflammation decreases cortical BDNF expression and contributes to the occurrence of cognitive impairment in schizophrenia [60,61]. Furthermore, increased IL-1β was implicated in the development of cognitive deficits, especially in working memory, probably as a result of altered function of microglia and reduced BDNF production [62].

The lack of the above correlation in the clozapine-treated group implies that clozapine may alleviate the negative loop IL-1β-BDNF possibly through, e.g., reduced activity of microglial cells. Indeed, increased serum BDNF levels were reported following long-term clozapine treatment [63]. However, the mechanism underlying stimulatory effect of clozapine on BDNF and neurogenesis are not fully understood. Our findings demonstrate that, in the group of all patients, BDNF correlated positively with FasL, yet only at admission. Interestingly, other studies have shown that pro-BDNF, through the activation of the p75NTR receptor, in the absence of Trk receptor expression, may lead to apoptosis of neurons [20,64]. Although we did not measure the isoforms of BDNF, we carefully hypothesize that, during relapse, the pro-BDNF isoform would prevail and that the synthesis of FasL would increase. Furthermore, we propose that the observed positive correlation between plasma BDNF and FasL levels may be linked with the predominance of the pro-BDNF form during the active phase of disease. It is tempting to postulate that an enhanced apoptosis, involving the pro-BDNF-FasL link and the p75NTR receptor, may characterize pharmaco-resistant schizophrenia. Indeed, the increase in pro-BDNF was recently demonstrated in patients at ultra-high risk of psychosis [65], and a higher pro-BDNF/m-BDNF ratio was shown in patients with first-episode schizophrenia [66]. Furthermore, the *BDNF* gene Val66Met polymorphism variation was linked to differences in attentional allocation to emotional faces. While this was found in a younger population, it gives support to the idea that BDNF-related neuroplasticity could influence emotional processing patterns across the lifespan [67]. It was recently shown that the *BDNF* gene rs6265 (C → T, Val → Met) polymorphism can be associated with the severity of affective and non-affective symptoms of schizophrenia. However, its role is not fully clarified, as epigenetic factors may modulate *BDNF* expression [68].

In light of these data, we may hypothesize that the positive correlation between peripheral levels of BDNF and FasL may reflect disease severity. Possibly, the disappearance of such correlation during treatment could indicate the resolution of an acute episode of the disease and confirm the beneficial effects of the therapy. Furthermore, prospective studies on a larger cohort of patients are needed to clarify this issue.

We propose that the active phase of psychosis may be associated with an imbalance between BDNF forms, with a higher proportion of the pro-BDNF isoform. The absence of a correlation between BDNF and FasL after 4-week therapy and during remission may in part result from the stimulatory effect of antipsychotics on neurotrophic signaling. Neuroleptic therapy may increase the secretion of m-BDNF in the studied patients by increasing the expression of metalloproteinase 2 (MMP-2), which converts the pro-BDNF form into m-BDNF. Indeed, risperidone and haloperidol were shown to activate MMP-2 and normalize neurogenesis in various animal models of schizophrenia [69]. The precise mechanisms linking antipsychotics with changes in apoptotic pathways require further research.

## 5. Conclusions

In agreement with previous works, the presented data support the notion that a pro-inflammatory environment is an important factor contributing to schizophrenia development. The severity of psychopathology in patients with schizophrenia seems associated with higher levels of IL-1β and lower levels of BDNF. The novelty of our findings is the observation that the higher level of FasL is negatively associated with the degree of clinical improvement in general symptoms. Thus, it may be postulated that a decline in FasL level during treatment may indicate the degree of clinical improvement in schizophrenia. Furthermore, we report that, in patients diagnosed with pharmaco-resistant schizophrenia and treated with clozapine, the negative correlations between IL-1β-BDNF and between the FasL level and leukocyte count are absent.

The presented results indirectly support the concept of an enhanced apoptotic processes in the course of schizophrenia. In the view of the available literature data and our findings, it is tempting to hypothesize that such increased apoptosis in patients with treatment-resistant schizophrenia may be linked with the pro-BDNF-FasL link. Although with caution, we propose that use of clozapine may be linked with a protective effect against FasL signaling and inhibition of apoptotic processes.

## 6. Limitations and Future Directions

The main limitation of this study is the relatively small number of participants in certain subgroups, particularly in the clozapine-treated group (*n* = 11), which may reduce statistical power and increase the risk of type II errors. While FDR correction confirmed all originally significant correlations, we note that the relatively small sample sizes in some subgroups limit statistical power, and the results should be interpreted as preliminary.

Future studies with larger and more balanced cohorts are needed to validate these exploratory findings. Furthermore, a prospective evaluation of the temporal pattern of the expression of BDNF isoforms, FasL, and other pro- and anti-apoptotic factors in patients diagnosed with schizophrenia is needed. Special attention should be directed to research on the potential correlations between clinical status and the above factors in patients suffering from treatment-resistant schizophrenia.

## Figures and Tables

**Table 1:** Study group characteristics with *p*-values for between-group comparisons.

Variable	Patient Group *n* (%) or Mean ± SD	Control Group *n* (%) or Mean ± SD	*p*-Value
Gender			
Male	34 (64.15)	21 (46.67)	0.08 ^a^
Female	19 (35.85)	24 (53.33)	>0.05
Total	53 (100.00)	45 (100.00)	>0.05
Education			
Primary	4 (7.55)	0 (0.00)	>0.05
Lower secondary	3 (5.66)	6 (13.33)	>0.05
Higher secondary	22 (41.51)	17 (37.78)	>0.05
Vocational training	4 (7.55)	0 (0.00)	>0.05
Higher (university)	17 (32.08)	22 (48.89)	>0.05
Total	53 (100.00)	45 (100.00)	>0.05
Continuous variables			
Age (years)	26.92 ± 8.18	24.17 ± 5.81	0.07 ^b^
Leukocytes (10^9^/L)	6.74 ± 1.92	6.03 ± 2.10	0.18 ^b^
BMI (kg/m^2^)	25.24 ± 4.76	22.14 ± 5.66	0.09 ^b^

The data are presented as mean values ± SD or values (%); no statistical differences were detected (Student’s *t*-test). ^a^ Chi-squared test for categorical variables. ^b^ Student’s *t*-test for independent samples.

**Table 2:** Disease variables in the studied patients.

Analyzed Variable	M	Me	Min	Max	Q1	Q3	SD
Time to first contact with a psychiatrist (months)	47.03	35	0	228	3	66	56.21
Prodromal period (months)	13.57	9	2	60	2	12	16.55
Duration of untreated psychosis (months)	11.42	6	1	48	2	12	13.86
Duration of the disease (months)	70.04	48	1	276	12	120	73.75
Number of disease exacerbations	2.94	2	1	11	1	4	2.51
Total number of hospitalizations	2.63	1	1	14	1	3	2.84

M—mean, Me—median, Min—minimum value, Max—maximum value, Q1—lower quartile, Q3—upper quartile, SD—standard deviation.

**Table 3:** PANSS, SAPS, and SANS scores, and plasma levels of IL-1β, BDNF, and FasL in schizophrenia patients at admission, after 4 weeks of treatment, and in remission.

Parameter	Admission	4 Weeks	*p* Value	Remission	*p* Value	Control
Clinical Schizophrenia Scales						
PANSS T	92.00 ± 21.7	**75.0 ± 20.7**	**<0.001**	**57.52 ± 20.6**	**<0.001**	N/A
PANSS P	21.90 ± 6.6	**17.2 ± 6.6**	**<0.001**	**11.90 ± 5.9**	**<0.001**	N/A
PANSS N	25.00 ± 7.3	**21.7 ± 6.7**	**<0.001**	**16.76 ± 6.6**	**<0.001**	N/A
PANSS G	45.00 ± 12.43	**36.7 ± 10.7**	**<0.001**	**28.79 ± 10.3**	**<0.001**	N/A
SAPS	42.90 ± 29.1	**32.1 ± 25.2**	**<0.001**	**15.40 ± 19.8**	**<0.001**	N/A
SANS	62.30 ± 23.6	**53.5 ± 21.4**	**<0.001**	**38.50 ± 21.3**	**<0.001**	N/A
IL-1β (pg/mL)						
All	1.75 ± 2.35	2.04 ± 3.71	>0.05	1.8 ± 1.92	>0.05	1.49 ± 1.16
Clozapine-treated	1.52 ± 2.15	1.86 ± 2.99	>0.05	1.5 ± 1.7	>0.05	N/A
Non-clozapine treatment	1.81 ± 2.42	3.21 ± 4.10	>0.05	1.96 ± 2.05	>0.05	N/A
FasL (pg/mL)						
All	78.94 ± 27.63	78.85 ± 24.95	>0.05	75.9 ± 23.52	>0.05	81.94 ± 34.74
Clozapine-treated	89.42 ± 34.98	82.10 ± 24.77	>0.05	80.02 ± 31.71	>0.05	N/A
Non-clozapine treatment	76.16 ± 25.24	77.31 ± 25.56	>0.05	73.95 ± 19.24	>0.05	N/A
BDNF (ng/mL)						
All	7.45 ± 5.22	6.74 ± 4.41	>0.05	7.83 ± 4.47	>0.05	5.85 ± 3.22
Clozapine-treated	6.83 ± 4.16	5.47 ± 4.08	>0.05	7.39 ± 3.98	>0.05	N/A
Non-clozapine treatment	7.62 ± 5.51	7.34 ± 4.54	>0.05	8.04 ± 4.77	>0.05	N/A

Data are shown as mean values ± SD. Clinical Schizophrenia Scales—statistical comparison vs. admission; Wilcoxon test. Due to extremely high significance and very low *p* value, it is denominated as <0.001. The Mann–Whitney U test was used for statistical comparisons of IL-1β, FasL, and BDNF levels with respective control values. N/A—not applicable. Statistically significant values in bold.

## Data Availability

The data are available on request due to ethical restrictions.

## Supplementary Materials

The following supporting information can be downloaded at: https://www.mdpi.com/article/10.3390/jcm14186417/s1, Table S1: Correlations of IL 1β with PANSS, SAPS, and SANS at admission, after 4 weeks, and in remission (with exact *p* values, 95% CI, FDR); Table S2: Correlations of IL-1β with cognitive test scores at admission, after 4 weeks, and in remission (exact *p*-values, 95% CI, FDR); Table S3: Correlations of FasL with PANSS, SAPS, and SANS at admission, after 4 weeks, and in remission (exact *p* values, 95% CI, FDR); Table S4: Correlations of FasL with cognitive test scores at admission, after 4 weeks, and in remission (exact *p*-values, 95% CI, FDR); Table S5: Correlations of BDNF with PANSS, SAPS, and SANS scores at admission, after 4 weeks of treatment, and in remission; Table S6: Correlations of BDNF with cognitive test scores at admission, after 4 weeks, and in remission (exact *p*-values, 95% CI, FDR); Table S7: Correlations between studied markers in schizophrenia patients at admission, after 4 weeks of treatment, and in remission; Table S8: PANSS, SAPS, and SANS scores correlations with IL-1β, FasL, and BDNF at admission, after 4 weeks of treatment, and in remission in patients with early-phase schizophrenia; Table S9: PANSS, SAPS, and SANS scores correlations with IL-1β, FasL, and BDNF at admission, after 4 weeks of treatment, and in remission in patients with chronic schizophrenia.

## Abbreviations

The following abbreviations are used in this manuscript:

BDNF	brain-derived neurotrophic factor
BMI	body mass index
BPRS	Brief Psychiatric Rating Scale/Brief Psychopathology Rating Scale
CSF	cerebrospinal fluid
CLO	clozapine-treated patients
ELISA	enzyme-linked immunosorbent assay
FADD	Fas-associated death domain
Fas	cluster of differentiation (CD) 95
FasL	Fas ligand
*FASLG*	Fas ligand gene
ICD	International Classification of Diseases
IFNα	interferon α
IL	interleukin
mBDNF	mature form of BDNF
MMP-2	metalloproteinase 2
NO-CLO	clozapine-naïve patients
NT-4	neurotrophin-4
p75NTR	p75 neurotrophin receptor
PANSS G	PANSS—general symptoms
PANSS N	PANSS—negative symptoms
PANSS P	PANSS—positive symptoms
PANSS T	PANSS total score
PANSS	Positive and Negative Symptom Scale for Schizophrenia
proBDNF	precursor form of BDNF
SANS	Scale for Assessment of Negative Symptoms
SAPS	Scale for Assessment of Positive Symptoms
TNF-α	tumor necrosis factor-α
Trk	tropomyosin receptor kinase
TrkB	tropomyosin receptor kinase B
