# Supplementary material for: Prognostic Value of FasL, BDNF, and IL-1β as Predictors of Therapeutic Response in Schizophrenia
Source: J Clin Med. 2025 Sep 11;14(18):6417. doi: 10.3390/jcm14186417 (PMC12470808; doi:10.3390/jcm14186417)
Supplement: Supplementary file 1 [file jcm-14-06417-s001.zip › jcm-3815430-supplementary.pdf]

SUPPLEMENTARY MATERIALS

Table S1. Correlations of IL-1β with PANSS, SAPS, and SANS at admission, after 4 weeks, and in remission (with exact p-values, 95% CI, FDR)

Parameter	Admission (r)	p-value	95% CI for r	4 weeks (r)	p-value	95% CI for r	Remission (r)	p-value	95% CI for r	Significant after FDR (q=0.05)
**IL-1β, All patients (N=53)**										
PANSS T	0.19	>0.05	—	0.27	>0.05	—	−0.04	>0.05	—	No
PANSS P	0.15	>0.05	—	0.20	>0.05	—	0.00	>0.05	—	No
**PANSS N**	**0.30**	**0.029**	**0.03 to 0.53**	0.38	>0.05	—	0.04	>0.05	—	Yes (adm)
PANSS G	0.15	>0.05	—	0.29	>0.05	—	−0.04	>0.05	—	No
SAPS	0.07	>0.05	—	0.31	>0.05	—	0.16	>0.05	—	No
**SANS**	0.26	>0.05	—	**0.44**	**0.0010**	**0.19 to 0.64**	0.17	>0.05	—	Yes (4w)
ΔPANSS T	−0.07	>0.05	—	−0.30	>0.05	—	−0.08	>0.05	—	No
**ΔPANSS P**	−0.16	>0.05	—	**−0.40**	**0.0030**	**−0.61 to −0.15**	−0.04	>0.05	—	Yes (4w)
ΔPANSS N	0.03	>0.05	—	−0.27	>0.05	—	0.05	>0.05	—	No
ΔPANSS G	0.01	>0.05	—	−0.21	>0.05	—	−0.19	>0.05	—	No
ΔSAPS	−0.14	>0.05	—	−0.30	>0.05	—	−0.02	>0.05	—	No
ΔSANS	−0.02	>0.05	—	0.07	>0.05	—	−0.03	>0.05	—	No
Number of relapses	−0.17	>0.05	—	0.01	>0.05	—	−0.07	>0.05	—	No
Leukocytes	−0.13	>0.05	—	0.15	>0.05	—	−0.14	>0.05	—	No
Duration of disease	−0.23	>0.05	—	−0.15	>0.05	—	0.13	>0.05	—	No
**IL-1β, Clozapine-treated (N=11)**										
PANSS T	−0.15	>0.05	—	−0.02	>0.05	—	−0.26	>0.05	—	No
PANSS P	−0.27	>0.05	—	0.01	>0.05	—	−0.08	>0.05	—	No
PANSS N	0.09	>0.05	—	0.33	>0.05	—	0.01	>0.05	—	No
PANSS G	−0.17	>0.05	—	0.02	>0.05	—	−0.30	>0.05	—	No
SAPS	−0.33	>0.05	—	−0.04	>0.05	—	0.20	>0.05	—	No
SANS	0.25	>0.05	—	0.23	>0.05	—	0.05	>0.05	—	No

**ΔPANSS T**	−0.45	>0.05	—	**−0.73**	**0.011**	**−0.93 to −0.23**	0.32	>0.05	—	Yes (4w)
**ΔPANSS P**	**−0.86**	**0.0006**	**−0.96 to −0.54**	**−0.83**	**0.0014**	**−0.96 to −0.46**	0.16	>0.05	—	Yes (adm, 4w)
ΔPANSS N	−0.10	>0.05	—	−0.46	>0.05	—	0.55	>0.05	—	No
ΔPANSS G	−0.12	>0.05	—	−0.33	>0.05	—	0.38	>0.05	—	No
ΔSAPS	−0.39	>0.05	—	−0.40	>0.05	—	0.07	>0.05	—	No
ΔSANS	0.08	>0.05	—	0.36	>0.05	—	0.28	>0.05	—	No
Number of relapses	0.22	>0.05	—	0.25	>0.05	—	−0.18	>0.05	—	No
Leukocytes	0.02	>0.05	—	0.60	>0.05	—	0.50	>0.05	—	No
Duration of disease	0.09	>0.05	—	−0.06	>0.05	—	0.18	>0.05	—	No
**IL-1β, Non-clozapine (N=42)**										
PANSS T	0.35	>0.05	—	0.47	>0.05	—	0.06	>0.05	—	No
PANSS P	0.23	>0.05	—	0.41	>0.05	—	0.03	>0.05	—	No
PANSS N	0.32	>0.05	—	0.45	>0.05	—	0.06	>0.05	—	No
PANSS G	0.33	>0.05	—	0.51	>0.05	—	0.02	>0.05	—	No
SAPS	0.18	>0.05	—	0.62	>0.05	—	0.19	>0.05	—	No
SANS	0.30	>0.05	—	0.69	>0.05	—	0.27	>0.05	—	No
ΔPANSS T	−0.034	>0.05	—	−0.279	>0.05	—	−0.259	>0.05	—	No
ΔPANSS P	0.016	>0.05	—	−0.303	>0.05	—	−0.104	>0.05	—	No
ΔPANSS N	−0.062	>0.05	—	−0.414	>0.05	—	−0.08	>0.05	—	No
ΔPANSS G	−0.077	>0.05	—	−0.21	>0.05	—	−0.45	>0.05	—	No
ΔSAPS	−0.089	>0.05	—	−0.29	>0.05	—	−0.06	>0.05	—	No
ΔSANS	−0.096	>0.05	—	0.01	>0.05	—	−0.28	>0.05	—	No
Number of relapses	−0.329	>0.05	—	−0.028	>0.05	—	−0.023	>0.05	—	No
Leukocytes	−0.252	>0.05	—	0.152	>0.05	—	−0.219	>0.05	—	No
**Duration of disease**	**−0.413**	**0.0066**	**−0.64 to −0.13**	−0.163	>0.05	—	0.044	>0.05	—	Yes (adm)

Table S2. Correlations of IL-1β with cognitive test scores at admission, after 4 weeks, and in remission (exact p-values, 95% CI, FDR)

**Parameter**	**Admission (r)**	**p-value**	**95% CI for r**	**4 weeks (r)**	**p-value**	**95% CI for r**	**Remission (r)**	**p-value**	**95% CI for r**	**Significant after FDR (q=0.05)**
**IL-1β, All patients (N=53)**										
TMT-A	−0.05	>0.05	—	−0.07	>0.05	—	−0.10	>0.05	—	No
TMT-B	−0.04	>0.05	—	−0.08	>0.05	—	−0.11	>0.05	—	No
Stroop (word)	−0.12	>0.05	—	−0.16	>0.05	—	−0.14	>0.05	—	No
Stroop (color)	−0.10	>0.05	—	−0.15	>0.05	—	−0.13	>0.05	—	No
Stroop (interf.)	−0.14	>0.05	—	−0.17	>0.05	—	−0.15	>0.05	—	No
Verbal fluency	0.08	>0.05	—	0.10	>0.05	—	0.11	>0.05	—	No
Digit span forward	0.05	>0.05	—	0.07	>0.05	—	0.09	>0.05	—	No
Digit span backward	0.07	>0.05	—	0.09	>0.05	—	0.08	>0.05	—	No
RAVLT immediate	0.09	>0.05	—	0.12	>0.05	—	0.14	>0.05	—	No
**RAVLT delayed**	**0.31**	**0.023**	**0.04 to 0.54**	0.29	>0.05	—	0.27	>0.05	—	Yes (adm)
**RAVLT recognition**	**0.33**	**0.015**	**0.06 to 0.55**	0.30	>0.05	—	0.29	>0.05	—	Yes (adm)
**IL-1β, Clozapine-treated (N=11)**										
TMT-A	−0.15	>0.05	—	−0.20	>0.05	—	−0.22	>0.05	—	No
TMT-B	−0.17	>0.05	—	−0.24	>0.05	—	−0.25	>0.05	—	No
Stroop (word)	−0.13	>0.05	—	−0.21	>0.05	—	−0.23	>0.05	—	No
Stroop (color)	−0.12	>0.05	—	−0.22	>0.05	—	−0.24	>0.05	—	No
Stroop (interf.)	−0.14	>0.05	—	−0.23	>0.05	—	−0.26	>0.05	—	No
Verbal fluency	0.16	>0.05	—	0.21	>0.05	—	0.22	>0.05	—	No
Digit span forward	0.14	>0.05	—	0.18	>0.05	—	0.19	>0.05	—	No

Digit span backward	0.15	>0.05	—	0.20	>0.05	—	0.21	>0.05	—	No
RAVLT immediate	0.12	>0.05	—	0.15	>0.05	—	0.17	>0.05	—	No
**RAVLT delayed**	**0.64**	**0.033**	**0.07 to 0.90**	0.59	>0.05	—	0.55	>0.05	—	Yes (adm)
**RAVLT recognition**	**0.66**	**0.028**	**0.10 to 0.91**	0.60	>0.05	—	0.57	>0.05	—	Yes (adm)
**IL-1β, Non-clozapine (N=42)**										
TMT-A	−0.06	>0.05	—	−0.08	>0.05	—	−0.09	>0.05	—	No
TMT-B	−0.07	>0.05	—	−0.09	>0.05	—	−0.10	>0.05	—	No
Stroop (word)	−0.13	>0.05	—	−0.15	>0.05	—	−0.14	>0.05	—	No
Stroop (color)	−0.11	>0.05	—	−0.13	>0.05	—	−0.13	>0.05	—	No
Stroop (interf.)	−0.12	>0.05	—	−0.14	>0.05	—	−0.14	>0.05	—	No
Verbal fluency	0.07	>0.05	—	0.09	>0.05	—	0.10	>0.05	—	No
Digit span forward	0.05	>0.05	—	0.07	>0.05	—	0.09	>0.05	—	No
Digit span backward	0.06	>0.05	—	0.08	>0.05	—	0.09	>0.05	—	No
RAVLT immediate	0.08	>0.05	—	0.10	>0.05	—	0.11	>0.05	—	No
**RAVLT delayed**	**0.29**	**0.032**	**0.03 to 0.51**	0.27	>0.05	—	0.26	>0.05	—	Yes (adm)
**RAVLT recognition**	**0.31**	**0.025**	**0.05 to 0.53**	0.29	>0.05	—	0.28	>0.05	—	Yes (adm)

Table S3. Correlations of FasL with PANSS, SAPS, and SANS at admission, after 4 weeks, and in remission (exact p-values, 95% CI, FDR)

Parameter	Admission (r)	p-value	95% CI for r	4 weeks (r)	p-value	95% CI for r	Remission (r)	p-value	95% CI for r	Significant after FDR (q=0.05)
**FasL, All patients (N=53)**										
PANSS T	0.14	>0.05	—	0.17	>0.05	—	0.12	>0.05	—	No
PANSS P	0.02	>0.05	—	0.07	>0.05	—	0.01	>0.05	—	No
PANSS N	0.15	>0.05	—	0.03	>0.05	—	0.06	>0.05	—	No
PANSS G	0.09	>0.05	—	0.15	>0.05	—	0.09	>0.05	—	No
SAPS	0.10	>0.05	—	0.15	>0.05	—	−0.06	>0.05	—	No
SANS	−0.08	>0.05	—	−0.12	>0.05	—	−0.00	>0.05	—	No
ΔPANSS T	−0.28	>0.05	—	−0.37	>0.05	—	−0.06	>0.05	—	No
ΔPANSS P	−0.05	>0.05	—	−0.30	>0.05	—	0.02	>0.05	—	No
ΔPANSS N	−0.12	>0.05	—	−0.09	>0.05	—	−0.10	>0.05	—	No
**ΔPANSS G**	**−0.39**	**0.0039**	**−0.60 to −0.13**	**−0.43**	**0.0013**	**−0.63 to −0.18**	0.04	>0.05	—	Yes (adm, 4w)
ΔSAPS	−0.29	>0.05	—	−0.19	>0.05	—	−0.16	>0.05	—	No
ΔSANS	0.01	>0.05	—	−0.17	>0.05	—	−0.14	>0.05	—	No
Number of relapses	−0.15	>0.05	—	−0.11	>0.05	—	0.03	>0.05	—	No
**Leukocyte count**	**−0.33**	**0.015**	**−0.55 to −0.07**	0.01	>0.05	—	−0.17	>0.05	—	Yes (adm)
Duration of disease	−0.06	>0.05	—	0.06	>0.05	—	−0.18	>0.05	—	No
**FasL, Clozapine-treated (N=11)**										
PANSS T	0.55	>0.05	—	0.66	>0.05	—	0.31	>0.05	—	No
PANSS P	0.55	>0.05	—	0.66	>0.05	—	0.50	>0.05	—	No
PANSS N	0.48	>0.05	—	−0.34	>0.05	—	0.06	>0.05	—	No
PANSS G	0.61	>0.05	—	0.64	>0.05	—	0.28	>0.05	—	No
SAPS	0.41	>0.05	—	0.64	>0.05	—	0.07	>0.05	—	No

SANS	0.16	>0.05	—	−0.19	>0.05	—	0.11	>0.05	—	No
**ΔPANSS T**	−0.37	>0.05	—	**−0.68**	**0.021**	**−0.91 to −0.13**	−0.60	>0.05	—	Yes (4w)
ΔPANSS P	0.38	>0.05	—	−0.43	>0.05	—	−0.26	>0.05	—	No
ΔPANSS N	−0.31	>0.05	—	−0.37	>0.05	—	−0.64	>0.05	—	No
**ΔPANSS G**	**−0.77**	**0.0050**	**−0.94 to −0.32**	**−0.70**	**0.017**	**−0.92 to −0.17**	−0.55	>0.05	—	Yes (adm, 4w)
ΔSAPS	−0.33	>0.05	—	0.17	>0.05	—	−0.42	>0.05	—	No
ΔSANS	0.13	>0.05	—	−0.28	>0.05	—	−0.65	>0.05	—	No
Number of relapses	0.58	>0.05	—	0.11	>0.05	—	−0.26	>0.05	—	No
Leukocyte count	−0.03	>0.05	—	−0.30	>0.05	—	0.00	>0.05	—	No
Duration of disease	0.13	>0.05	—	0.02	>0.05	—	−0.55	>0.05	—	No
**FasL, Non-clozapine (N=42)**										
PANSS T	−0.06	>0.05	—	−0.01	>0.05	—	−0.28	>0.05	—	No
PANSS P	−0.20	>0.05	—	−0.31	>0.05	—	−0.42	>0.05	—	No
PANSS N	0.09	>0.05	—	0.04	>0.05	—	−0.31	>0.05	—	No
PANSS G	−0.15	>0.05	—	−0.06	>0.05	—	−0.12	>0.05	—	No
SAPS	−0.11	>0.05	—	−0.13	>0.05	—	−0.28	>0.05	—	No
SANS	−0.14	>0.05	—	−0.05	>0.05	—	−0.18	>0.05	—	No
ΔPANSS T	−0.08	>0.05	—	−0.17	>0.05	—	0.50	>0.05	—	No
ΔPANSS P	−0.02	>0.05	—	−0.11	>0.05	—	0.36	>0.05	—	No
ΔPANSS N	0.10	>0.05	—	0.08	>0.05	—	0.36	>0.05	—	No
ΔPANSS G	−0.16	>0.05	—	−0.30	>0.05	—	0.54	>0.05	—	No
ΔSAPS	−0.22	>0.05	—	−0.37	>0.05	—	0.08	>0.05	—	No
ΔSANS	0.13	>0.05	—	−0.03	>0.05	—	0.20	>0.05	—	No
Number of relapses	−0.33	>0.05	—	−0.16	>0.05	—	0.17	>0.05	—	No

**Leukocyte count**	**−0.43**	**0.0045**	**−0.65 to −0.15**	0.07	>0.05	—	−0.14	>0.05	—	Yes (adm)
Duration of disease	−0.21	>0.05	—	0.09	>0.05	—	−0.03	>0.05	—	No

Table S4. Correlations of FasL with cognitive test scores at admission, after 4 weeks, and in remission (exact p-values, 95% CI, FDR)

**Parameter**	**Admission (r)**	**p-value**	**95% CI for r**	**4 weeks (r)**	**p-value**	**95% CI for r**	**Remission (r)**	**p-value**	**95% CI for r**	**Significant after FDR (q=0.05)**
**FasL, All patients (N=53)**										
TMT-A	0.04	>0.05	—	0.06	>0.05	—	0.05	>0.05	—	No
TMT-B	0.05	>0.05	—	0.07	>0.05	—	0.06	>0.05	—	No
Stroop (word)	0.06	>0.05	—	0.08	>0.05	—	0.07	>0.05	—	No
Stroop (color)	0.07	>0.05	—	0.09	>0.05	—	0.08	>0.05	—	No
Stroop (interf.)	0.08	>0.05	—	0.10	>0.05	—	0.09	>0.05	—	No
Verbal fluency	−0.06	>0.05	—	−0.07	>0.05	—	−0.06	>0.05	—	No
Digit span forward	−0.05	>0.05	—	−0.06	>0.05	—	−0.05	>0.05	—	No
Digit span backward	−0.04	>0.05	—	−0.05	>0.05	—	−0.04	>0.05	—	No
RAVLT immediate	−0.07	>0.05	—	−0.08	>0.05	—	−0.07	>0.05	—	No
RAVLT delayed	−0.09	>0.05	—	−0.10	>0.05	—	−0.09	>0.05	—	No
**RAVLT recognition**	**−0.32**	**0.020**	**−0.54 to −0.06**	−0.30	>0.05	—	−0.28	>0.05	—	Yes (adm)
**FasL, Clozapine-treated (N=11)**										
TMT-A	0.12	>0.05	—	0.15	>0.05	—	0.14	>0.05	—	No
TMT-B	0.13	>0.05	—	0.16	>0.05	—	0.15	>0.05	—	No
Stroop (word)	0.14	>0.05	—	0.17	>0.05	—	0.16	>0.05	—	No
Stroop (color)	0.15	>0.05	—	0.18	>0.05	—	0.17	>0.05	—	No
Stroop (interf.)	0.16	>0.05	—	0.19	>0.05	—	0.18	>0.05	—	No
Verbal fluency	−0.17	>0.05	—	−0.19	>0.05	—	−0.18	>0.05	—	No
Digit span forward	−0.18	>0.05	—	−0.20	>0.05	—	−0.19	>0.05	—	No
Digit span backward	−0.19	>0.05	—	−0.21	>0.05	—	−0.20	>0.05	—	No

RAVLT immediate	−0.20	>0.05	—	−0.22	>0.05	—	−0.21	>0.05	—	No
RAVLT delayed	−0.21	>0.05	—	−0.23	>0.05	—	−0.22	>0.05	—	No
**RAVLT recognition**	**−0.68**	**0.022**	**−0.91 to −0.13**	−0.65	>0.05	—	−0.63	>0.05	—	Yes (adm)
**FasL, Non-clozapine (N=42)**										
TMT-A	0.03	>0.05	—	0.05	>0.05	—	0.04	>0.05	—	No
TMT-B	0.04	>0.05	—	0.06	>0.05	—	0.05	>0.05	—	No
Stroop (word)	0.05	>0.05	—	0.07	>0.05	—	0.06	>0.05	—	No
Stroop (color)	0.06	>0.05	—	0.08	>0.05	—	0.07	>0.05	—	No
Stroop (interf.)	0.07	>0.05	—	0.09	>0.05	—	0.08	>0.05	—	No
Verbal fluency	−0.08	>0.05	—	−0.09	>0.05	—	−0.08	>0.05	—	No
Digit span forward	−0.07	>0.05	—	−0.08	>0.05	—	−0.07	>0.05	—	No
Digit span backward	−0.06	>0.05	—	−0.07	>0.05	—	−0.06	>0.05	—	No
RAVLT immediate	−0.09	>0.05	—	−0.10	>0.05	—	−0.09	>0.05	—	No
RAVLT delayed	−0.10	>0.05	—	−0.11	>0.05	—	−0.10	>0.05	—	No
**RAVLT recognition**	**−0.30**	**0.042**	**−0.54 to −0.01**	−0.28	>0.05	—	−0.27	>0.05	—	Yes (adm)

Table S5. Correlations of BDNF with PANSS, SAPS, and SANS scores at admission, after 4 weeks of treatment, and in remission.

Parameter	Admission	p-value	4 weeks	p-value	Remission	p-value
**BDNF (ng/ml), All patients**						
**Clinical Schizophrenia Scale**						
PANSS T	0.03	>0.05	-0.29	>0.05	0.09	>0.05
PANSS P	0.04	>0.05	-0.09	>0.05	0.04	>0.05
PANSS N	0.00	>0.05	-0.53	>0.05	-0.04	>0.05
PANSS G	-0.01	>0.05	-0.19	>0.05	0.09	>0.05
SAPS	0.09	>0.05	-0.23	>0.05	0.17	>0.05
SANS	0.05	>0.05	-0.35	>0.05	0.05	>0.05
**Degree of clinical improvement**						
ΔPANSS T	-0.18	>0.05	0.21	>0.05	-0.15	>0.05
ΔPANSS P	-0.04	>0.05	0.11	>0.05	0.13	>0.05
ΔPANSS N	-0.10	>0.05	0.28	>0.05	-0.17	>0.05
ΔPANSS G	-0.33	>0.05	0.06	>0.05	-0.28	>0.05
ΔSAPS	-0.25	>0.05	-0.17	>0.05	-0.26	>0.05
ΔSANS	-0.06	>0.05	0.10	>0.05	-0.03	>0.05
**Variable**						
Number of relapses	0.08	>0.05	0.14	>0.05	0.02	>0.05
Leukocyte count	0.02	>0.05	0.17	>0.05	-0.58	>0.05
Duration of the disease	0.13	>0.05	0.24	>0.05	-0.04	>0.05
**BDNF (ng/ml), Clozapine-treated**						
**Clinical Schizophrenia Scale**						
PANSS T	0.31	>0.05	0.02	>0.05	0.15	>0.05
PANSS P	0.47	>0.05	-0.01	>0.05	-0.10	>0.05
PANSS N	-0.04	>0.05	-0.47	>0.05	-0.30	>0.05
PANSS G	0.44	>0.05	0.19	>0.05	0.20	>0.05
SAPS	0.62	>0.05	0.47	>0.05	-0.05	>0.05
SANS	-0.15	>0.05	0.04	>0.05	-0.26	>0.05
**Degree of clinical improvement**						
ΔPANSS T	0.05	>0.05	0.10	>0.05	0.03	>0.05
ΔPANSS P	0.17	>0.05	-0.25	>0.05	0.14	>0.05
ΔPANSS N	-0.23	>0.05	-0.05	>0.05	-0.30	>0.05
ΔPANSS G	-0.18	>0.05	-0.07	>0.05	-0.35	>0.05
ΔSAPS	-0.38	>0.05	-0.52	>0.05	-0.20	>0.05
ΔSANS	-0.13	>0.05	-0.33	>0.05	0.13	>0.05
**Variable**						
Number of relapses	0.17	>0.05	0.15	>0.05	-0.26	>0.05
Leukocytes	0.09	>0.05	-0.30	>0.05	0.00	>0.05
Duration of the disease	0.03	>0.05	0.18	>0.05	-0.41	>0.05
**BDNF (ng/ml), Non-clozapine treatment**						
**Clinical Schizophrenia Scale**						
PANSS T	-0.07	>0.05	-0.41	>0.05	0.21	>0.05
PANSS P	-0.10	>0.05	-0.13	>0.05	0.16	>0.05
PANSS N	0.06	>0.05	-0.54	>0.05	0.06	>0.05
PANSS G	-0.12	>0.05	-0.32	>0.05	0.09	>0.05
SAPS	-0.10	>0.05	-0.47	>0.05	0.23	>0.05
SANS	0.12	>0.05	-0.44	>0.05	0.11	>0.05

**Degree of clinical improvement**						
ΔPANSS T	-0.16	>0.05	0.16	>0.05	-0.01	>0.05
ΔPANSS P	-0.02	>0.05	0.27	>0.05	0.27	>0.05
ΔPANSS N	0.01	>0.05	0.28	>0.05	-0.02	>0.05
ΔPANSS G	-0.34	>0.05	0.05	>0.05	-0.11	>0.05
ΔSAPS	-0.23	>0.05	-0.05	>0.05	-0.12	>0.05
ΔSANS	0.01	>0.05	0.31	>0.05	0.01	>0.05
**Variable**						
Number of relapses	0.09	>0.05	0.07	>0.05	0.14	>0.05
Leukocytes	0.06	>0.05	0.22	>0.05	-0.67	>0.05
Duration of the disease	0.14	>0.05	0.38	>0.05	0.19	>0.05

Data are shown as a correlation coefficient (r); Spearman correlations.

Table S6. Correlations of BDNF with cognitive test scores at admission, after 4 weeks, and in remission (exact p-values, 95% CI, FDR)

**Parameter**	**Admission (r)**	**p-value**	**95% CI for r**	**4 weeks (r)**	**p-value**	**95% CI for r**	**Remission (r)**	**p-value**	**95% CI for r**	**Significant after FDR (q=0.05)**
**BDNF, All patients (N=53)**										
TMT-A	−0.04	>0.05	—	−0.05	>0.05	—	−0.06	>0.05	—	No
TMT-B	−0.05	>0.05	—	−0.06	>0.05	—	−0.07	>0.05	—	No
Stroop (word)	−0.06	>0.05	—	−0.07	>0.05	—	−0.08	>0.05	—	No
Stroop (color)	−0.07	>0.05	—	−0.08	>0.05	—	−0.09	>0.05	—	No
Stroop (interf.)	−0.08	>0.05	—	−0.09	>0.05	—	−0.10	>0.05	—	No
Verbal fluency	0.05	>0.05	—	0.06	>0.05	—	0.07	>0.05	—	No
Digit span forward	0.04	>0.05	—	0.05	>0.05	—	0.06	>0.05	—	No
Digit span backward	0.03	>0.05	—	0.04	>0.05	—	0.05	>0.05	—	No
**RAVLT immediate**	**0.33**	**0.016**	**0.06 to 0.55**	0.31	>0.05	—	0.30	>0.05	—	Yes (adm)
**RAVLT delayed**	**0.35**	**0.010**	**0.09 to 0.57**	0.33	>0.05	—	0.32	>0.05	—	Yes (adm)
**RAVLT recognition**	**0.36**	**0.0086**	**0.10 to 0.58**	0.34	>0.05	—	0.33	>0.05	—	Yes (adm)
**BDNF, Clozapine-treated (N=11)**										
TMT-A	−0.15	>0.05	—	−0.16	>0.05	—	−0.17	>0.05	—	No
TMT-B	−0.16	>0.05	—	−0.17	>0.05	—	−0.18	>0.05	—	No
Stroop (word)	−0.17	>0.05	—	−0.18	>0.05	—	−0.19	>0.05	—	No
Stroop (color)	−0.18	>0.05	—	−0.19	>0.05	—	−0.20	>0.05	—	No
Stroop (interf.)	−0.19	>0.05	—	−0.20	>0.05	—	−0.21	>0.05	—	No
Verbal fluency	0.16	>0.05	—	0.17	>0.05	—	0.18	>0.05	—	No
Digit span forward	0.15	>0.05	—	0.16	>0.05	—	0.17	>0.05	—	No

Digit span backward	0.14	>0.05	—	0.15	>0.05	—	0.16	>0.05	—	No
**RAVLT immediate**	**0.69**	**0.019**	**0.15 to 0.92**	0.66	>0.05	—	0.64	>0.05	—	Yes (adm)
**RAVLT delayed**	**0.71**	**0.015**	**0.19 to 0.93**	0.68	>0.05	—	0.66	>0.05	—	Yes (adm)
**RAVLT recognition**	**0.73**	**0.012**	**0.23 to 0.94**	0.70	>0.05	—	0.68	>0.05	—	Yes (adm)
**BDNF, Non-clozapine (N=42)**										
TMT-A	−0.05	>0.05	—	−0.06	>0.05	—	−0.07	>0.05	—	No
TMT-B	−0.06	>0.05	—	−0.07	>0.05	—	−0.08	>0.05	—	No
Stroop (word)	−0.07	>0.05	—	−0.08	>0.05	—	−0.09	>0.05	—	No
Stroop (color)	−0.08	>0.05	—	−0.09	>0.05	—	−0.10	>0.05	—	No
Stroop (interf.)	−0.09	>0.05	—	−0.10	>0.05	—	−0.11	>0.05	—	No
Verbal fluency	0.04	>0.05	—	0.05	>0.05	—	0.06	>0.05	—	No
Digit span forward	0.03	>0.05	—	0.04	>0.05	—	0.05	>0.05	—	No
Digit span backward	0.02	>0.05	—	0.03	>0.05	—	0.04	>0.05	—	No
**RAVLT immediate**	**0.31**	**0.028**	**0.04 to 0.54**	0.29	>0.05	—	0.28	>0.05	—	Yes (adm)
**RAVLT delayed**	**0.33**	**0.019**	**0.06 to 0.55**	0.31	>0.05	—	0.30	>0.05	—	Yes (adm)
**RAVLT recognition**	**0.34**	**0.015**	**0.07 to 0.56**	0.32	>0.05	—	0.31	>0.05	—	Yes (adm)

Table S7. Correlations between studied markers in schizophrenia patients at admission, after 4 weeks of treatment, and in remission.

**Parameter**	**Admission**	**p-value**	**4 weeks**	**p-value**	**Remission**	**p-value**
IL -1β (pg/ml), All patients						
BDNF (ng/ml)	-0.08	>0.05	**-0.48**	**0.012**	0.23	>0.05
IL -1β (pg/ml), Clozapine-treated						
BDNF (ng/ml)	-0.63	>0.05	-0.22	>0.05	-0.3	>0.05
IL -1β (pg/ml), Non-clozapine treatment						
BDNF (ng/ml)	0.15	>0.05	**-0.57**	**0.013**	0.41	>0.05
FasL (pg/ml), All patients						
BDNF (ng/ml)	**0.34**	**0.025**	0.07	>0.05	-0.01	>0.05
FasL (pg/ml), Clozapine-treated						
BDNF (ng/ml)	0.53	>0.05	0.3	>0.05	0.13	>0.05
FasL (pg/ml), Non-clozapine treatment						
BDNF (ng/ml)	0.25	>0.05	-0.01	>0.05	-0.05	>0.05

Data are shown as a correlation coefficient (r); Spearman correlations.

Table S8. PANSS, SAPS, and SANS scores correlations with IL-1β, FasL, and BDNF at admission, after 4 weeks of treatment, and in remission in patients with early-phase schizophrenia.

**Parameter**	**Admission**	**p-value**	**4 weeks**	**p-value**	**Remission**	**p-value**
**IL -1β (pg/ml)**						
**Clinical Schizophrenia Scale**						
PANSS T	0.39	>0.05	0.61	>0.05	-0.24	>0.05
PANSS P	0.00	>0.05	0.33	>0.05	-0.27	>0.05
PANSS N	0.43	>0.05	**0.83**	**0,04**	-0.33	>0.05
PANSS G	0.43	>0.05	0.61	>0.05	-0.05	>0.05
SAPS	0.04	>0.05	0.32	>0.05	0.14	>0.05
SANS	0.41	>0.05	**0.73**	**0,05**	-0.19	>0.05
**Degree of clinical improvement**						
ΔPANSS T	0.10	>0.05	-0.60	>0.05	-0.11	>0.05
ΔPANSS P	-0.02	>0.05	-0.74	>0.05	-0.19	>0.05
ΔPANSS N	0.26	>0.05	-0.25	>0.05	0.02	>0.05
ΔPANSS G	0.08	>0.05	**-0.72**	**0.04**	-0.22	>0.05
ΔSAPS	0.10	>0.05	-0.11	>0.05	0.02	>0.05
ΔSANS	0.48	>0.05	-0.32	>0.05	0.11	>0.05
**Variable**						
Number of relapses	0.14	>0.05	0.00	>0.05	-0.18	>0.05
Leukocytes	-0.07	>0.05	-0.50	>0.05	0.40	>0.05
Duration of the disease	0.05	>0.05	0.31	>0.05	0.08	>0.05
**FasL (pg/ml)**						
**Clinical Schizophrenia Scale**						
PANSS T	-0.09	>0.05	0.17	>0.05	0.00	>0.05
PANSS P	-0.27	>0.05	-0.05	>0.05	-0.60	>0.05
PANSS N	-0.01	>0.05	-0.22	>0.05	0.12	>0.05
PANSS G	-0.02	>0.05	0.17	>0.05	-0.12	>0.05
SAPS	-0.28	>0.05	0.02	>0.05	-0.47	>0.05
SANS	-0.44	>0.05	-0.19	>0.05	-0.11	>0.05

**Degree of clinical improvement**						
ΔPANSS T	0.28	>0.05	-0.15	>0.05	-0.19	>0.05
ΔPANSS P	0.28	>0.05	-0.48	>0.05	-0.24	>0.05
ΔPANSS N	0.19	>0.05	-0.18	>0.05	-0.20	>0.05
ΔPANSS G	0.42	>0.05	-0.07	>0.05	0.00	>0.05
ΔSAPS	0.55	>0.05	0.12	>0.05	-0.29	>0.05
ΔSANS	0.00	>0.05	-0.52	>0.05	-0.18	>0.05
**Variable**						
Number of relapses	0.32	>0.05	-0.17	>0.05	0.33	>0.05
Leukocytes	-0.48	>0.05	0.00	>0.05	-0.40	>0.05
Duration of the disease	0.34	>0.05	0.47	>0.05	-0.14	>0.05
**BDNF (ng/ml)**						
**Clinical Schizophrenia Scale**						
PANSS T	-0.06	>0.05	-0.60.	>0.05	0.18	>0.05
PANSS P	-0.20	>0.05	-0.39	>0.05	0.22	>0.05
PANSS N	0.11	>0.05	-0.66.	>0.05	-0.19	>0.05
PANSS G	-0.10	>0.05	-0.60.	>0.05	0.05	>0.05
SAPS	0.36	>0.05	-0.52	>0.05	0.25	>0.05
SANS	0.41	>0.05	**-0.79**	**0,03**	0.34	>0.05
**Degree of clinical improvement**						
ΔPANSS T	-0.13	>0.05	0.67	>0.05	-0.69	>0.05
ΔPANSS P	-0.22	>0.05	0.50	>0.05	-0.62	>0.05
ΔPANSS N	-0.11	>0.05	0.58	>0.05	-0.54	>0.05
ΔPANSS G	-0.18	>0.05	**0.78**	**0,03**	-0.74	>0.05
ΔSAPS	-0.28	>0.05	0.48	>0.05	-0.54	>0.05
ΔSANS	-0.32	>0.05	0.40	>0.05	-0.68	>0.05
**Variable**						
Number of relapses	0.59	>0.05	0.39	>0.05	-0.52	>0.05
Leukocytes	0.59	>0.05	0.80	>0.05	0.80	>0.05

Duration of the disease	-0.35	>0.05	-0.02	>0.05	0.30	>0.05

Data are shown as a correlation coefficient (r); Spearman correlations.

Table S9. PANSS, SAPS, and SANS scores correlations with IL-1β, FasL, and BDNF at admission, after 4 weeks of treatment, and in remission in patients with chronic schizophrenia.

**Parameter**	**Admission**	**p-value**	**4 weeks**	**p-value**	**Remission**	**p-value**
**IL -1β (pg/ml)**						
**Clinical Schizophrenia Scale**						
PANSS T	-0.09	>0.05	0.27	>0.05	-0.07	>0.05
PANSS P	-0.01	>0.05	0.12	>0.05	0.19	>0.05
PANSS N	0.09	>0.05	0.62	>0.05	0.01	>0.05
PANSS G	-0.20	>0.05	0.16	>0.05	-0.21	>0.05
SAPS	-0.16	>0.05	0.18	>0.05	0.29	>0.05
SANS	-0.08	>0.05	0.61	>0.05	0.03	>0.05
**Degree of clinical improvement**						
ΔPANSS T	-0.11	>0.05	-0.35	>0.05	**-0.82**	**0,015**
ΔPANSS P	-0.30	>0.05	0.05	>0.05	-0.65	>0.05
ΔPANSS N	0.19	>0.05	-0.07	>0.05	-0.64.	>0.05
ΔPANSS G	-0.17	>0.05	-0.35	>0.05	**-0.90**	**0,001**
ΔSAPS	-0.12	>0.05	-0.36	>0.05	-0.63.	>0.05
ΔSANS	-0.21	>0.05	0.17	>0.05	-0.30	>0.05
**Variable**						
Number of relapses	-0.05	>0.05	0.64	>0.05	-0.77	>0.05
Leukocytes	0.02	>0.05	-0.21	>0.05	-0.50	>0.05
Duration of the disease	0.22	>0.05	-0.24	>0.05	-0.49	>0.05
**FasL (pg/ml)**						
**Clinical Schizophrenia Scale**						
PANSS T	0.28	>0.05	0.18	>0.05	0.29	>0.05
PANSS P	0.12	>0.05	0.13	>0.05	0.58	>0.05
PANSS N	0.40	>0.05	-0.05	>0.05	0.15	>0.05
PANSS G	0.26	>0.05	0.23	>0.05	0.34	>0.05
SAPS	0.12	>0.05	0.14	>0.05	0.51	>0.05
SANS	0.19	>0.05	-0.61	>0.05	0.13	>0.05

**Degree of clinical improvement**						
ΔPANSS T	**-0.75**	**0.005**	**-0.72**	**0,02**	-0.34	>0.05
ΔPANSS P	-0.44	>0.05	-0.39	>0.05	0.00	>0.05
ΔPANSS N	-0.31	>0.05	0.01	>0.05	-0.13	>0.05
ΔPANSS G	**-0.78**	**0.003**	**-0.91**	**0.0001**	-0.34	>0.05
ΔSAPS	-0.53	>0.05	-0.29	>0.05	-0.54	>0.05
ΔSANS	**-0.62**	**0.044**	0.00	>0.05	-0.33	>0.05
**Variable**						
Number of relapses	-0.46	>0.05	-0.41	>0.05	-0.26	>0.05
Leukocytes	-0.21	>0.05	0.10	>0.05	-0.50	>0.05
Duration of the disease	-0.28	>0.05	-0.34	>0.05	-0.74	>0.05
**BDNF (ng/ml)**						
**Clinical Schizophrenia Scale**						
PANSS T	-0.05	>0.05	**-0.75**	**0,02**	0.15	>0.05
PANSS P	0.10	>0.05	-0.55	>0.05	-0.13	>0.05
PANSS N	-0.23	>0.05	-0.38	>0.05	0.30	>0.05
PANSS G	-0.11	>0.05	**-0.69**	**0,03**	-0.05	>0.05
SAPS	0.10	>0.05	-0.71	>0.05	-0.05	>0.05
SANS	-0.21	>0.05	0.04	>0.05	0.10	>0.05
**Degree of clinical improvement**						
ΔPANSS T	-0.01	>0.05	0.30	>0.05	-0.12	>0.05
ΔPANSS P	0.16	>0.05	0.01	>0.05	0.00	>0.05
ΔPANSS N	0.01	>0.05	0.13	>0.05	-0.37	>0.05
ΔPANSS G	-0.13	>0.05	0.58	>0.05	0.09	>0.05
ΔSAPS	-0.50	>0.05	0.14	>0.05	0.12	>0.05
ΔSANS	-0.42	>0.05	-0.55	>0.05	-0.18	>0.05
**Variable**						
Number of relapses	0.70	>0.05	0.64	>0.05	0.04	>0.05
Leukocytes	0.00	>0.05	-0.26	>0.05	1.00	>0.05

Duration of the disease	0.28	>0.05	**0.74**	**0,014**	**0.70**	**0,05**

Data are shown as a correlation coefficient (r); Spearman correlations.
